# Effects of Brain Size on Adult Neurogenesis in Shrews

**DOI:** 10.3390/ijms22147664

**Published:** 2021-07-17

**Authors:** Katarzyna Bartkowska, Krzysztof Turlejski, Beata Tepper, Leszek Rychlik, Peter Vogel, Ruzanna Djavadian

**Affiliations:** 1 Nencki Institute of Experimental Biology Polish Academy of Sciences, 02-093 Warsaw, Poland; k.bartkowska@nencki.edu.pl (K.B.); b.tepper@nencki.edu.pl (B.T.); 2 Faculty of Biology and Environmental Sciences, Cardinal Stefan Wyszynski University in Warsaw, 01-938 Warsaw, Poland; k.turlejski@uksw.edu.pl; 3 Department of Systematic Zoology, Institute of Environmental Biology, Adam Mickiewicz University, 61-712 Poznan, Poland; rychliklesz@gmail.com; 4 Department of Ecology and Evolution, University of Lausanne, 1015 Lausanne, Switzerland; peter.vogel@unil.ch

**Keywords:** shrews, rostral migratory stream, olfactory bulb, hippocampal neurogenesis, doublecortin, BrdU

## Abstract

Shrews are small animals found in many different habitats. Like other mammals, adult neurogenesis occurs in the subventricular zone of the lateral ventricle (SVZ) and the dentate gyrus (DG) of the hippocampal formation. We asked whether the number of new generated cells in shrews depends on their brain size. We examined *Crocidura russula* and *Neomys fodiens*, weighing 10–22 g, and *Crocidura olivieri* and *Suncus murinus* that weigh three times more. We found that the density of proliferated cells in the SVZ was approximately at the same level in all species. These cells migrated from the SVZ through the rostral migratory stream to the olfactory bulb (OB). In this pathway, a low level of neurogenesis occurred in *C. olivieri* compared to three other species of shrews. In the DG, the rate of adult neurogenesis was regulated differently. Specifically, the lowest density of newly generated neurons was observed in *C. russula*, which had a substantial number of new neurons in the OB compared with *C. olivieri*. We suggest that the number of newly generated neurons in an adult shrew’s brain is independent of the brain size, and molecular mechanisms of neurogenesis appeared to be different in two neurogenic structures.

## 1. Introduction

Shrews are small eutherian mammals found in Eurasia, Africa and both Americas. A total of 461 living shrew species of different metabolism and habits are classified into the family Soricidae, encompassing three extant subfamilies; Crocidurinae, Myosoricinae and Soricinae. Although shrews are generally small animals, the difference in body mass between species is huge. The smallest representative of shrews is *Suncus etruscus*, weighing 1.8–3 g, while among the largest representative is *Suncus* murinus, weighing up to 147.3 g [1,2]. Accordingly, the smallest species have the small brain mass, and larger animals have larger brains. However, the brain and body mass relationship is not linear between different mammalian species, and in addition, not always the larger brain manifests in evolutionary progress [3,4]. On the other hand, brains of larger species have more neurons than smaller brains within each mammalian order [5]. The cellular scaling rules have been identified to describe changes in brain structures for different mammalian species. These cellular rules were applied for rodents and primates [6,7]. According to this, larger brains have more neurons, smaller neuronal densities and larger glia/neuron ratios than smaller brains.

Shrews of the genus *Sorex* change their brain and body size in response to environmental changes, which is known as Dehnel’s effect. This effect was first described in *Sorex minutus* and has since been found in *Sorex araneus* living in various geographical regions [8,9]. During the summer–winter period, the braincase of *S. araneus* decreases by 15–20%, while shrinkage of brain mass is in the range of 10–26% [10,11,12]. Mechanisms that lead to seasonal changes in brain mass are not completely clear. Bartkowska et al. [13] showed that seasonal changes in brain mass do not depend on recruitment of new neurons or removal of existing cells, including adult neurogenesis. 

In mammals, adult neurogenesis occurs in the subventricular zone of the lateral ventricle (SVZ) and subgranular zone of the dentate gyrus (DG), known as hippocampal adult neurogenesis [14,15,16,17]. Meta-analysis of adult hippocampal neurogenesis between mammalian orders showed that there are some variations between laboratory and wild species of mammals [18]. The main difference was that adult hippocampal neurogenesis is more stable in wild animals, although such research in wild animals is scarce. With attempts to understand adult neurogenesis in brains of different species of shrews, we hypothesize that adult neurogenesis in small and large brains should be positively or negatively correlated with brain size.

Here, we analyzed the correlation of brain size and adult neurogenesis in shrews belonging to the order Eulipotyphla. We examined adult neurogenesis in both small shrews [representatives were the greater white-toothed shrew (*Crocidura russula*) and the Eurasian water shrew (*Neomys fodiens*)] and large shrews [representatives were the African giant shrew (*Crocidura olivieri*) and the Asian house shrew (*Suncus murinus*)]. 

## 2. Results

### 2.1. Body and Brain Masses

**Figure 1:**
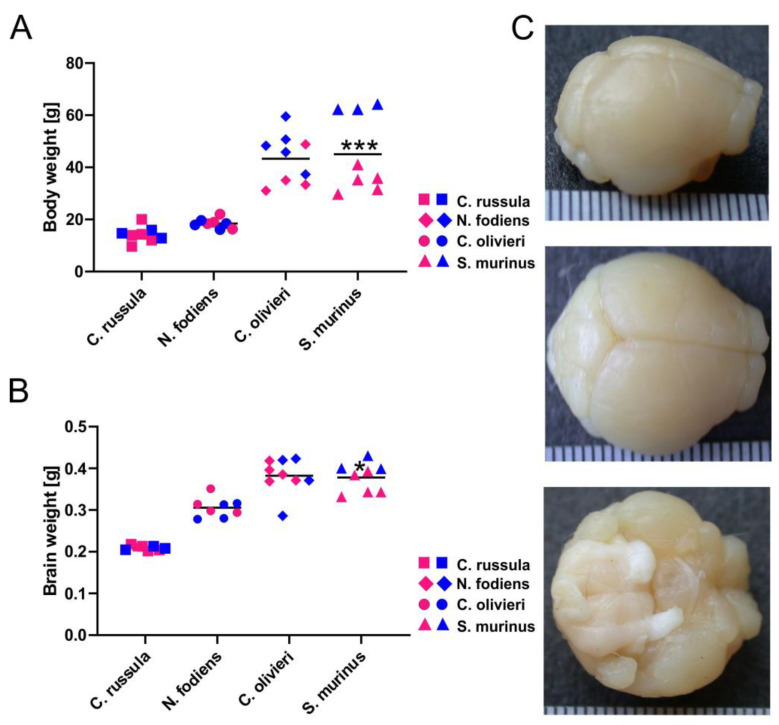
Body and brain mass of four different species of shrews; *C. russula* (squares), *N. fodiens* (circles), *C. olivieri* (rhombuses) and *S. murinus* (triangles). (**A**) Body mass of all investigated shrews. The blue and red symbols show the results for male and female shrews, respectively. (**B**) Brain mass of all investigated shrews. The blue and red symbols show the results for male and female shrews, respectively. (**C**) Lateral, dorsal and ventral views of the brain of *N. fodiens*. Scale: the distance between the lines is 500 µm. * *p* < 0.05; *** *p* < 0.001.

We analyzed body and brain masses of four different species of shrews in the family Soricidae (Supplementary Figure S1). Two species, *C. russula* and *N. fodiens*, had a relatively small body mass, and the remaining two species, *C. olivieri* and *S. murinus*, had a large body mass. The body mass of small shrews (*C. russula* (13.46 g ± 1.96) and *N. fodiens* (18.46 g ± 1.89)) was between 10–22 g, while the body mass of large species (*C. olivieri* (43.87 g ± 9.21) and *S. murinus* (45.03 g ± 14.99)) was three times more (Figure 1A). We also examined whether female and male body masses differ within species and found that *S. murinus* males were markedly heavier (62.67 g ± 1.15) than females (34.44 g ± 4.37), and this difference was significant (*p* < 0.0001). Conversely, *N. fodiens* females were slightly heavier in body mass than males; however, this difference was not significant. Age of all *N. fodiens* shrews was in the range of 9–12 months, except for three *N. fodiens* that were approximately 4–6 months old. We included the data of these young animals in our study, because we found no differences in body and brain masses of these and the remaining overwintered water shrews. 

Relatively small brain mass was found in *C. russula* (0.21 g ± 0.001) and *N. fodiens* (0.30 g ± 0.008), while the brain mass in *C. olivieri* and *S. murinus* was 0.38 g ± 0.01 (Figure 1B). We did not find any sex difference in brain mass within species with the exception of *S. murinus.* In this species, male brains were heavier than female brains. The brains of investigated shrews were almost round, and the neocortex was smooth (Figure 1C).

### 2.2. Proliferation and Migration of BrdU Labeled Nuclei

**Figure 2:**
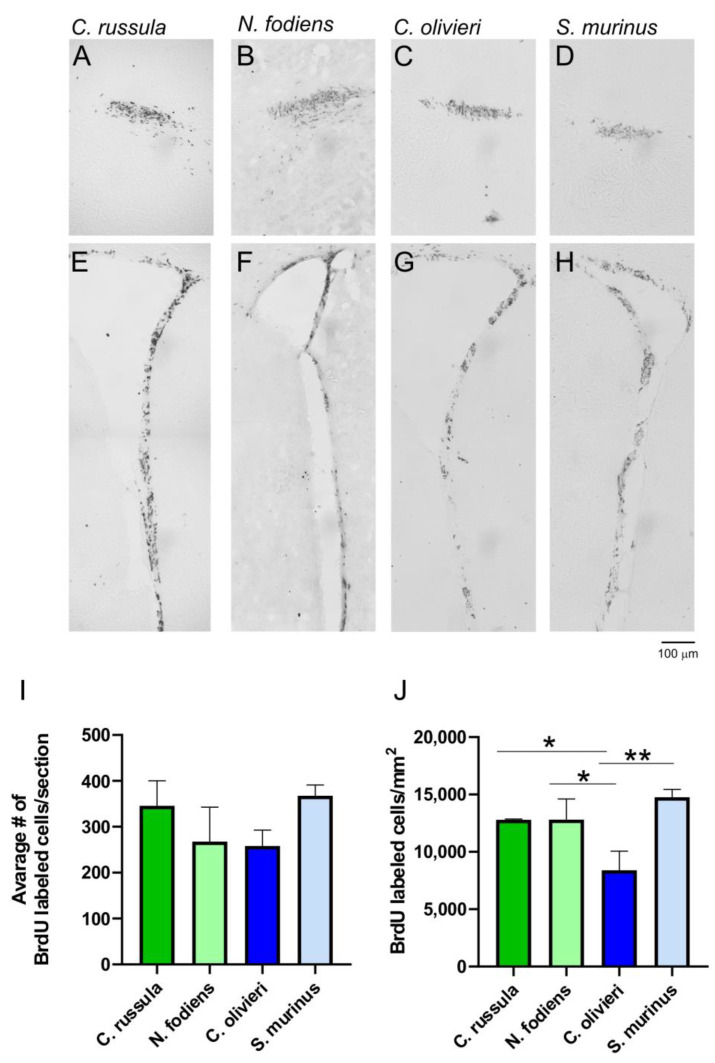
Localization and quantification of BrdU-labeled cells in the SVZ and RMS of the four studied shrew species. (**A**–**D**) Localization of BrdU-labeled cells in the RMS at the level of the SVZ. (**E**–**H**) Localization of BrdU-labeled cells in the SVZ. (**I**) The average number of BrdU immunopositive cells per section in the SVZ. (**J**) The numbers of BrdU immunopositive cells/mm^2^ in the RMS. * *p* < 0.05; ** *p* < 0.01; SVZ, subventricular zone of the lateral ventricle; RMS, rostral migratory stream.

To examine proliferation and the origin of newly generated neurons in the brains of adult shrews, animals were injected twice with BrdU at a 2 h interval. BrdU-labeled cells were detected after 2 days of animals’ treatment using immunohistochemistry. The majority of labeled cells were visible in the SVZ, where they were densely packed in the subependymal layer (Figure 2E–H). The nuclei of BrdU-labeled cells were elongated and small. Although there was no significant difference in the numbers of proliferated cells between investigated species of shrews (F(3,9) = 1.28, *p* = 0.33), the density of BrdU-labeled cells was the lowest in *C. olivieri* (Figure 2I). The newly generated cells started to migrate, and many of them, 48 h after BrdU treatment, were localized towards the rostral migratory stream (RMS) (Figure 2A–D). A few BrdU-positive cells were also placed in the inner part of the OB granule layer. The density of BrdU immunopositive cells was less in the RMS of *C. olivieri* than in other species (Figure 2J), and a one way ANOVA showed a significant difference in this main effect (F(3,9) = 4.25, *p* = 0.039). The Fisher’s LSD multiple comparisons test revealed a significant difference between *C. olivieri* vs. *C. russula* (*p* = 0.045), *C. olivieri* vs. *N. fodiens* (*p* = 0.044) and *C. olivieri* vs. *S. murinus* (*p* = 0.008).

**Figure 3:**
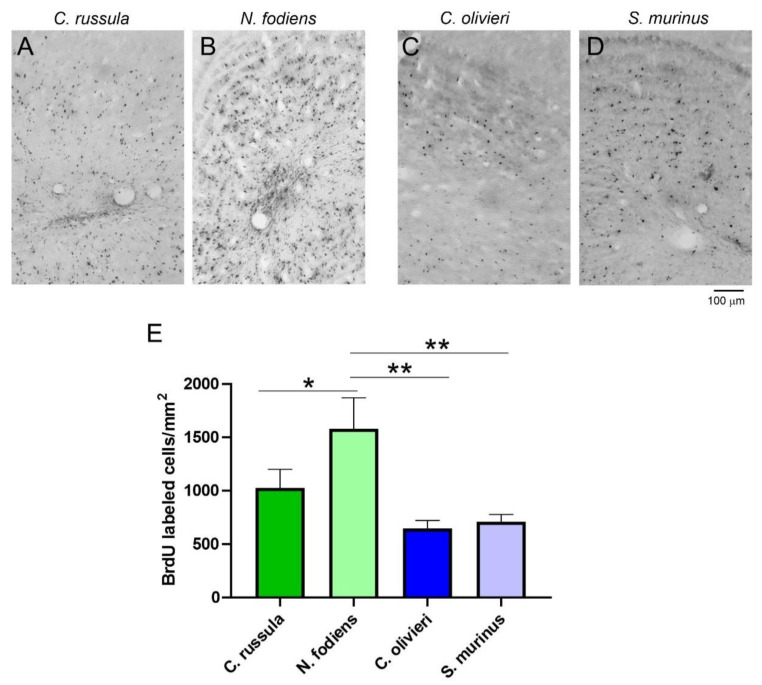
Localization and quantification of BrdU-labeled cells in the OB of the four shrew species. (**A**–**D**) Distribution of BrdU-positive cells in the OB. (**E**) The number of BrdU-labeled cells/mm^2^ in different species of shrews. OB, olfactory bulb, * *p* < 0.05, ** *p* < 0.01.

Next, we investigated migration and final placement of newly generated neurons. For this study, animals were perfused 2 weeks after BrdU injections. We found that most of the newly generated cells were present in the granule layer of the OB (Figure 3A–D), although a small population of BrdU-labeled cells was observed in the glomerular layer of the OB and the SVZ. Quantification of these data showed that there was a main effect of the number of BrdU-positive cells (Figure 3E). A one way ANOVA revealed a significant difference in the number of BrdU-labeled cells in the OB of various shrews that were perfused 14 days later after BrdU treatment (F (3, 16) = 5.88, *p* = 0.007). The Fisher’s LSD multiple comparisons test revealed a significant difference in the density of BrdU-labeled cells of the OB between *N. fodiens* and *C. russula* (*p* = 0.04), *N. fodiens* and *C. olivieri* (*p* = 0.002) and *N. fodiens* and *S. murinus* (*p* = 0.003).

**Figure 4:**
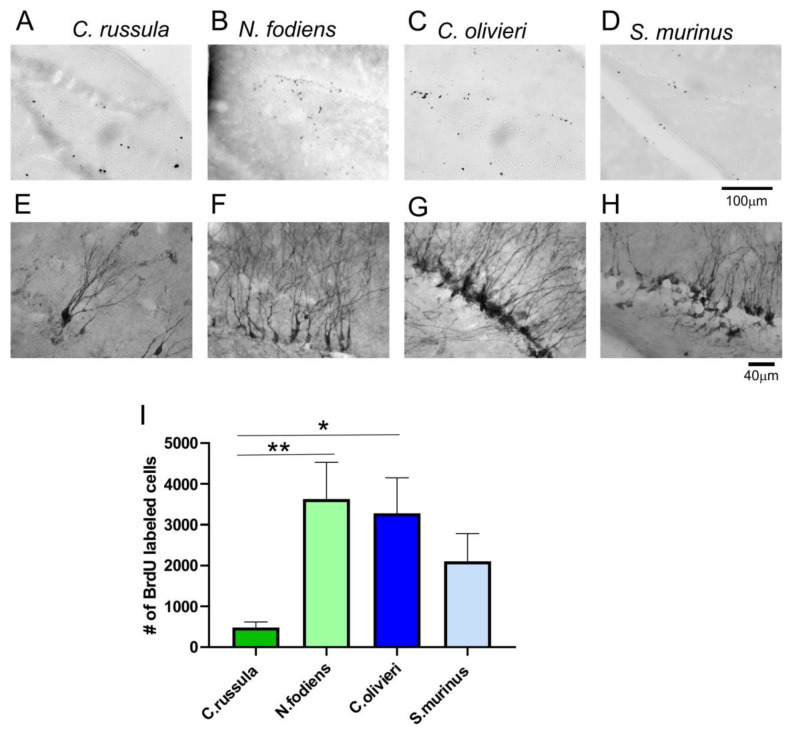
Localization and quantification of BrdU-labeled cells in the DG of investigated shrews. (**A**–**D**) Distribution of BrdU immunopositive cells in the DG of four investigated species of shrews. (**E**–**H**) Localization of DCX-positive cells in the DG. (**A**,**C**,**D**,**E**,**G**,**H**) Images obtained from adult shrews. (**B**,**F**) Images obtained from subadult *N. fodiens* shrew. (**I**) Mean numbers of BrdU-positive cells in the DG of shrews. DG, dentate gyrus, * *p* < 0.05, ** *p* < 0.01.

In the second neurogenic structure, 2 days after BrdU administration, labeled cells were visible in the subgranular layer of the DG, which is a place for proliferation of progenitor cells (Figure 4A–D). The visualized nuclei were small, round or slightly elongated. In the DG of *C. russula*, the number of BrdU-labeled cells was the lowest, and accordingly, the mean total number of BrdU-labeled cells in the DG was 480 vs. 3276 in *C. olivieri*. We defined that in the groups with shorter survival times after BrdU injection, more BrdU-labeled cells were identified than in animals that were perfused 14 days after BrdU injections (Figure 4I). A one way ANOVA showed that there was a significant difference in the main effect between species (F(3,16) = 3.93, *p* = 0.028). The smallest population of BrdU-labeled cells in the DG was present in *C. russula* (Figure 4I). DCX labeled cells were also sparse in the DG of *C. russula* (Figure 4E–H).

### 2.3. Neural Differentiation 

**Figure 5:**
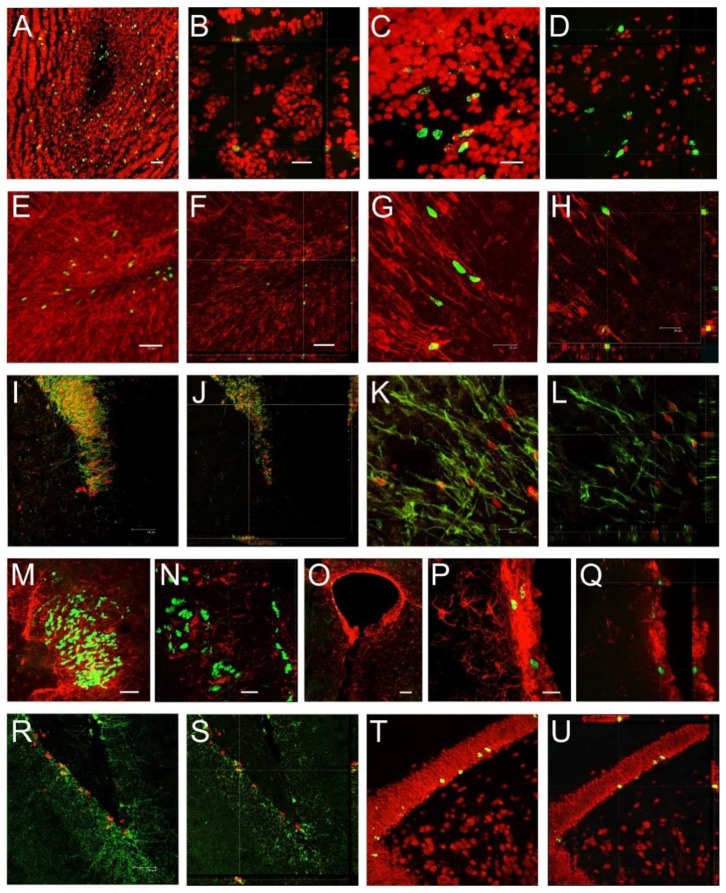
Detection of newly generated neurons using a double immunohistochemistry technique. (**A**–**D**) BrdU (green) and NeuN (red).Shrews were perfused 14 days after BrdU injections. (**E**–**H**) Double labeling with BrdU (green) and DCX (red) in the OB of a shrew (*C. olivieri*) that was perfused 2 days after BrdU administration. (**I**–**L**) Localization of BrdU (red)- and DCX (green)-labeled cells in the RMS of a shrew (*S. murinus*) at the level of SVZ (**I**,**J**) and OB (**K**,**L**). (**M**–**G**) Localization of BrdU (green)- and GFAP (red)-labeled cells in the RMS of a shrew (*N. fodiens*) at the level of SVZ (**M**,**N**) and SVZ (**O**–**Q**). (**R**,**S**) BrdU (red)- and DCX (green)-labeling in the DG of a shrew (*C. russula*) and (**T,U**) BrdU (green) and NeuN (red) of a shrew (*N. fodiens*) that was perfused 14 days after BrdU treatment. (**B**,**F**,**H**,**J**,**L**,**Q**,**S**,**U**) Higher magnification of orthogonal confocal images. The scale bar in (**A**) equals 50 µm and refers to (**O**). The scale bar in (**B**) equals 20 µm and refers to (**D**,**H**,**L**). The scale bar in (**C**) equals 20 µm and refers to (**G**,**K**). The scale bar in (**E**) equals 50 µm and refers to (**I**,**R**,**T**). The scale bar in (**F**) equals 50 µm and refers to (**J**,**S**,**U**). The scale bar in (**M**) equals 50 µm. The scale bar in (**N**) equals 20 µm and refers to (**Q**). The scale bar in (**P**) equals 20 µm.

To examine the further fate of cells incorporating BrdU, we used a double fluorescent technique, immunostaining BrdU-labeled cells with NeuN, a neuronal marker, DCX, which is a marker of neuronal progenitors and very young neurons, or GFAP, a marker for astrocytes. In animals perfused 14 days after BrdU injections, approximately 95% of granule cells in the OB were double-labeled with NeuN and BrdU (Figure 5A,B), whereas in the RMS at the level of the OB, no BrdU-positive cells colocalized with NeuN (Figure 5C,D). In the RMS at both levels of OB (Figure 5E–H) and SVZ (Figure 5I,J), 80–85% of BrdU-labeled cells had colocalization with DCX. In animals 2 days after BrdU injections, colocalization between BrdU and DCX was also observed in cells that reached the OB (Figure 5K,L). Additionally, in the RMS, we found no colocalization between BrdU and GFAP (Figure 5M,N), while BrdU-labeled cells located in the SVZ colocalized with GFAP (Figure 5O–Q). Approximately 40% of BrdU-positive cells colocalized with DCX (Figure 5R,S) or NeuN in the DG (Figure 5T,U). In the DG of animals that survived 2 days after BrdU treatment, a large number (95%) of DCX-labeled cells were localized in the granular layer, while most of BrdU immunopositive cells were placed in the subgranular layer.

### 2.4. Association between Adult Neurogenesis and Brain Mass

**Figure 6:**
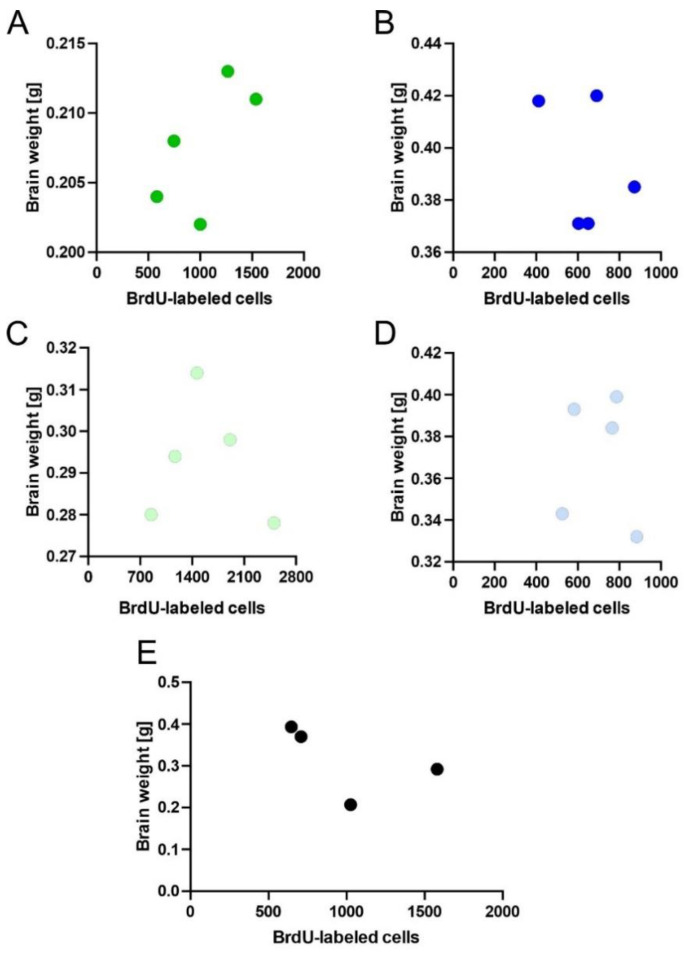
Analysis of the relationship between the number of BrdU-labeled cells and brain mass data. (**A**–**D**) Pearson correlation analyses between neurogenesis and brain mass in *C. russula* (**A**), *C. olivieri* (**B**), *N. fodiens* (**C**) and *S. murinus* (**D**). (**E**) A Pearson correlation of adult neurogenesis and brain weight in all investigated species of shrews.

To calculate the association between the number of BrdU-positive cells and brain mass, we analyzed a linear dependence between these two variables. The density of BrdU-incorporated cells in the OB or DG was not correlated with the size of the brain within species of shrews. Pearson correlation analyses established that there was no significant relationship (*p* = 0.44) between the number of BrdU-positive cells and brain mass within a selected shrew species (Figure 6A–D). Furthermore, we found no correlation between the number of BrdU-labeled cells and brain mass in investigated species of shrews (Figure 6E).

## 3. Discussion

We have demonstrated that in four investigated species of shrews, after 2 days of BrdU administration, the number of newly generated cells in the SVZ and RMS was very high, with the exception of *C. olivieri* that had a low number of proliferated cells in the RMS. The proportion of BrdU-labeled cells in various brain structures was changed in animals that were perfused 14 days after BrdU administration. In these animals, the majority of new cells were located in the OB, and the highest number of new neurons was observed in *N. fodiens* that we classified as a small-mass animal. In another species, which was also considered as a small-mass animal, *C. russula*, the number of BrdU-labeled cells was similar to that of large-weight animals. In the second neurogenic brain region, the DG, the number of BrdU-labeled cells was relatively small. Interestingly, the lowest rate of neurogenesis was estimated in the DG of *C. russula*, which had a high level of neurogenesis in the RMS and the OB. 

A number of papers have described the relationship between brain and body size [19,20,21], but it is still widely debated how brain mass varies with body size. In the present study, we reported a relationship between brain and body size in four species of the order Eulipotyphla, which is the third largest eutherian order after Rodentia and Chiroptera. The investigated animals live in diverse geographical locations (Europe (*N. fodiens*, *C. russula*), Asia (*S. murinus*) and Africa (*C. olivieri*)) and environments (tropical rain forest, savannah (*C. olivieri*), households (*S. murinus*), dry forest coasts (*C. russula*), damp meadows and bush adjoining water (*N. fodiens*)). Furthermore, all of them have a similar anatomy and diet [22]. Body and brain size change during development and reach a steady state when animals are adults. Therefore, the age of animals is an essential factor for comparisons between different species of animals. In our research, two species of shrews, *C. olivieri* and *S. murinus*, were bred at the University of Lausanne and treated with BrdU at an age of 7–9-months-old. Another species of shrews, *C. russula*, was captured during the winter as a juvenile and kept for four months in the animal house. Accordingly, they were injected with BrdU at the age of 9–11 months. The age of *N.fodiens* shrews was defined based on morphological features. Five of these shrews were adults (ages 9–12 months), except for three *N. fodiens* shrews that were not overwintered. However, their body and brain masses did not differ from that of overwintered animals, and they were considered as subadults and analyzed as the remaining individuals of this group. We also focused on sex differences and found that *S. murinus* shrews exhibited male-biased sexual dimorphism. Males’ body mass was almost twice that of females, while only a 15% difference was observed in brain mass between S. murinus males and females. The other two species, *N. fodiens* and *C. olivieri*, appeared to be similar based on sex. In both species, sex-related variables were not found, including body and brain masses. Interestingly, in the fourth investigated species, *C. russula*, some females were larger than males, but the difference in mean body masses between the sexes was insignificant. Our data are consistent with previous reports [23,24], apart from controversial data demonstrating sexual dimorphism in the body mass of *N. fodiens* [25]. Additionally, our findings suggest that in three species of shrews (*N. fodiens*, *C. russula* and *C. olivieri*), the sex factor is not involved in the regulation of brain size.

During phylogenetic development, the brain size of mammalian species increases with body size, with the only exception to this being the comparison of wild and domesticated animals of the same species. Commonly, wild animals have a smaller body mass and relatively bigger brain volume than their domesticated counterparts. This has been shown in European wild boars vs. pigs, wolves vs. dogs [26], and in wild and laboratory rat strains [27]. In smaller mammals, brain mass constitutes a higher proportion of body mass, which has important ecological consequences, as the resting metabolism of the brain is higher than that of other tissues. We examined the body–brain relationship in four species of shrews, representative of two subfamilies (Soricinae and Crocidurinae). Soricinae are characterized by a very high metabolism, and they do not fall into daily torpor or hibernation, while the metabolism of Crocidurinae is lower, and they fall into torpor during the day, lowering their body temperature to 22–24 °C [28]. As a consequence, Soricinae are able to survive in very cold environments, while Crocidurinae generally live in warmer habitats. Our data showed that the brain mass of small and large animals of both subfamilies correlates with the body mass. Neither the geographical location nor the environment influenced the body–brain relationship of the investigated shrews. 

Generally, larger brains have lower neuronal densities and a higher glia/neuron ratio [29]. The proportion of glial cells increases with brain size across rodent species, but to a lesser extent across primate species, and it does not vary significantly with brain size across insectivores [30]. This corresponds to results showing a scarcity of GFAP expression in the brain of *Sorex minutus* [31]. As neurons consume much more energy than glia [32], the brain of smaller species is an even more metabolically costly organ than in the large species. Moreover, proportionally larger brains do not always result in improved cognition that gives the species an evolutionary advantage, compensating for their higher metabolic cost [4,28].

In mammals, including shrews, adult neurogenesis occurs in two neurogenic areas. New cells are continuously generated in the SVZ and migrate rostrally to the OB, where they are incorporated into neuronal circuits. Stem cells proliferated in the second neurogenic area of the brain migrate a very short distance from the subgranular zone to the granular layer of the DG. Adult neurogenesis in both neurogenic brain structures is regulated by internal and external environmental factors, such as hormones, age, disease and many others [33,34,35,36,37,38,39]. One of the most essential factors modulating adult neurogenesis is brain aging. So far, available knowledge for adult neurogenesis during aging is consistent, which is that the rate of adult neurogenesis is reduced by 80–90% in all investigated animals, including primates [40,41]. Interestingly, in some echolocation bats and common shrews (*Sorex araneus)*, adult neurogenesis was present at low levels or absent throughout late life [13,18]. Newly generated neurons do not increase the cell population of the brain, because they incorporate into pre-existing circuitry where old neurons permanently die by apoptosis [42]. Therefore, adult neurogenesis does not change brain size, but brain size appears to regulate the rate of adult neurogenesis during phylogenetic development [43]. In our study, the age of *C. olivieri* and *S. murinus* was 7–9 months, while *C. russula* was approximately 2 months older. In nature, the reproducing cohort of *C. russula* may live as old as 18 months. Therefore*, C. russula* shrews were not considered old animals. Interestingly, the lowest rate of hippocampal neurogenesis was observed in *C. russula*, and we are inclined to think that the aging factor becomes more important in the hippocampal but not in the OB neurogenesis.

Many mammalian species exhibit male-biased sexual dimorphism, which means that, among other measures, brain size is larger in males than females. Sex differences in brain size were not observed in shrews within the species studied by us, except *S. murinus*. We found that female *S. murinus* brains weighed approximately 15% less than male brains. Indeed, difference in brain mass did not affect the rate of adult neurogenesis. Similar to our results, Lagace et al. [44] have demonstrated no sex differences in adult neurogenesis of C57BL/6 mice. However, there are some contradicting data concerning sex implication in the regulation of adult neurogenesis. A recent paper has reported that sex differences are present in the hippocampal adult neurogenesis of Sprague Dawley rats [45], confirming results of past research [46]. We found that among two adult water shrews (*N. fodiens*) that were captured at the same day, the number of BrdU-labeled cells was numerous in the DG of the female compared to the male shrew. Most likely, changes in the number of BrdU cells were caused by the sex hormones in these shrews. However, this group was too small to draw such a conclusion. 

Because hippocampal neurogenesis was found to correlate with hippocampal volume, we hypothesized that animal brain size might provide a correlate of neurogenesis within the order. We analyzed proliferation and dynamics of migration processes in two small-mass and two large-mass species of shrews. We found a lack of correlation between brain size and the number of newly proliferated cells in the SVZ through RMS to OB areas and the DG among different species of shrews. Interestingly, the generation process in two different neurogenic regions, the SVZ and DG, in investigated animals had a different pattern that is most likely mediated by different mechanisms. Our results show that *C. russula* had a similar number of newly generated cells in the OB as large-mass shrews, whereas levels of neurogenesis in the DG were very low. This indicates that molecular mechanisms of neurogenesis in shrews are different in two neurogenic structures.

## 4. Materials and Methods 

### 4.1. Animals

The research was performed on the following species of shrews: the greater white-toothed shrew (*Crocidura russula*, N = 8), the Asian house shrew (*Suncus murinus*, N = 8) and the African giant shrew (*Crocidura olivieri*, N = 9), bred and/or kept at the University of Lausanne, Switzerland under license to P. Vogel, and the Eurasian water shrew (*Neomys fodiens*, N = 8), trapped in Białowieża, Poland by K. Turlejski under license from the Polish Ministry of the Environment. All shrews were overwintered, sexually mature and classified as adult, except for three subadult *N. fodiends* that were not overwintered. *C. olivieri* and *S. murinus* were born from September to November at the University of Lausanne colony. After overwintering, they were injected with BrdU in June of the next year. Juvenile *C. russula* shrews were captured in February and kept for 4 months in the animal house at the University of Lausanne before BrdU injection. Only one species of shrew, *N. fodiens*, was captured in Poland in the period from May to September. Five of these shrews were classified as adults and injected with BrdU the following day, while three young shrews were kept for 2–2.5 months in the animal house in Białowieża and then injected with BrdU as subadults. All experimental procedures were approved by the State Committee for the Ethics of Animal Experimentation and were compatible with the standards of the Polish Law on Experimenting on Animals, which implemented the European Communities Council Directive of 24 November 1986 (86/609/EEC), as well as with the NIH Guide for the Care and Use of Laboratory Animals.

### 4.2. Bromodeoxyuridine (BrdU) Treatment and Brain Isolation

BrdU (Sigma-Aldrich, Darmstadt, Germany), a thymidine analogue, was given intraperitoneally, twice at 50 mg/kg in 0.9% NaCl, every 2 h to investigate changes in the rate of proliferation. Animals were weighed and injected with pentobarbital (200 mg/kg, i.p.) and perfused transcardially with 0.9% NaCl and then with 4% paraformaldehyde in 0.1M phosphate buffer. Three animals in each group, except *C. olivieri*, which was four animals, were perfused 2 days after BrdU injections, and five animals in each species were perfused 14 days after BrdU treatment. Brains were isolated from the skull and weighed immediately with an accuracy of 0.5 mg. Tissue was then post-fixed in 4% paraformaldehyde, cryoprotected with 30% sucrose and cut coronally on a cryostat into 40-µm sections. All sections of each brain were collected and arranged into 10 series, of which one was Nissl stained to allow for identification of brain structures. One randomly chosen series was used for BrdU or DCX labelling. The remaining series were used for double labeling or controls, or sometimes to confirm the results of the labelling.

### 4.3. BrdU Labelling

Free flowing 40 μm brain sections of these animals were washed in double concentrated saline-sodium citrate buffer (SSC). To increase tissue permeability, sections were incubated for 2 h in a 50% formamide solution in twice concentrated SSC at 60 °C. After 5 min of washing in SSC, the sections were denatured in 2 N HCl at 37 °C for 30 min and washed for 10 min in 0.1 M boric acid (pH 8.5). To block endogenous peroxidase, sections were soaked for 30 min in 3% H_2_O_2_ and 10% methyl alcohol in tris-buffered saline (TBS). Then, a series of sections was washed for 15 min in TBS with 0.1% Triton X-100 (TBS-A) and for 15 min in TBS-A with 0.05% BSA-bovine serum albumin (TBS-B). After blocking with 10% normal goat serum (NGS) in TBS-B, the sections were incubated overnight at 4 °C with a mouse monoclonal anti-BrdU antibody (1:1000, Roche, Basel, Switzerland) in TBS-B. Next, after washing in TBS-A and TBS-B, the sections were incubated for 45 min in a biotinylated anti-mouse antibody (1:200, Sigma-Aldrich, Darmstadt, Germany), followed by subsequent incubation in Extravidin (1:100, extravidin-biotin-peroxidase complex, Sigma-Aldrich, Darmstadt, Germany) in TBS. Peroxidase was detected by reaction with 0.05% 3,3′-diaminobenzidine chromium and 0.003% H_2_O_2_, in the presence of nickel (DAB Peroxidase Substrate Kit, Vector Laboratories). Finally, after several washes in TBS, the sections were applied to slides, dehydrated and finally covered. 

### 4.4. DCX Labeling 

One series of free-flowing brain sections was used for immunostaining. Sections were washed three times in phosphate buffered saline (PBS), pH 7.4, to remove the cryoprotective solution. Endogenous peroxidase was then blocked by PBS with 0.05% Triton X-100 (PBST), 10% H_2_O_2_ and 1% methyl alcohol for 30 min. The sections were washed three times in PBST and then incubated in 10% NGS and 0.1% BSA for 2 h. Next, the mouse anti-DCX antibody (1:500, Santa Cruz Biotechnology, Heidelberg, Germany) was applied overnight at 4 °C. The following day, sections were washed and incubated for 1.5 h in biotin-conjugated secondary polyclonal anti-rabbit antibody (1:300, Vector Laboratories, Burlingame, CA, USA). Sections were washed three times in PBST and incubated in PBS with StreptAvidine (1:400, Vector Laboratories, Burlingame, CA, USA). The color reaction was induced with horseradish peroxidase substrate, 3,3′-diaminobenzidine chromium and 0.05% H_2_O_2_ (DAB Peroxidase Substrate Kit, Vector Laboratories, Burlingame, CA, USA). The sections were washed three times in phosphate buffer and then placed on gelatinized slides, dehydrated and coversliped with DePeX mounting medium (Serva, Electrophoresis GmbH, Heidelberg, Germany). 

### 4.5. Double Fluorescence Staining

The sections were washed and permeabilized for BrdU as described above. Then, primary antibodies, mouse anti-BrdU (1:1000, Roche, Basel, Switzerland) and rabbit anti-NeuN (1:20, Cell Signaling Technology Europe, Leiden, The Netherlands) or rabbit anti-GFAP (1:500, DAKO, Agilent Tehnologies, Santa Clara, CA, USA) or goat anti- DCX (1:100, Santa Cruz Biotechnology, Heidelberg, Germany), were applied at 4 °C. After overnight incubation, sections were washed in PBST and then incubated with mixed secondary antibodies, conjugated with fluorochrome, anti-mouse 488 and anti-rabbit 568, anti-mouse 568 and anti-rabbit 488 or anti-goat 488 and anti-mouse 568 (1:600, Alexa Fluor, Thermo Fisher Scientific, Waltham, MA, USA). Finally, the sections were coverslipped with a mounting medium. 

### 4.6. Data Analysis

BrdU immunolabeled brain sections were imaged and analyzed using a Nikon Eclipse 90i microscope with the camera connected to a computer with Neurolucida (MBF Bioscience, Williston, VT, USA) or ImagePro software. To quantify the number of BrdU-positive cells in the SVZ and RMS, all labeled cells were counted on three brain sections using Image Fiji software. The numbers of labeled neurons in both hemispheres on the three sections were added and divided by the structure numbers. The number of BrdU-positive cells in the DG were counted in one series of every 10 coronal sections. The total number of BrdU-labeled cells was multiplied by 10 [13].

The density of BrdU immunopositive cells was estimated counting six OB sections. Each OB was sampled into the four anterior, posterior, ventral and dorsal counting areas. The average counting area for *C. russula*, *N. fodiens*, *C. olivieri* and *S. murinus* was 0.206013 mm^2^, 0.150392 mm^2^, 0.216912 mm^2^ and 0.215327 mm^2^, respectively. The number of BrdU labeled cells was counted using Fiji software that allows to count cells in the total depth of 40-μm sections. Afterwards, the density of labeled (cells/mm^2^) was calculated. This manual counting method was adapted from Li et al. [47]. 

Pictures from double immunofluorescent sections were captured and analyzed using a spinning disc confocal laser microscope (Zeiss, Jena, Germany). The data were analyzed using a one-way analysis of variance (ANOVA) followed by the post hoc Fisher’s least significant difference (LSD) analysis using GraphPad Prism software. Differences were considered significant for *p* < 0.05.

## Data Availability

The data of the current study are available from the corresponding author on request.

## Supplementary Materials

The following are available online at https://www.mdpi.com/article/10.3390/ijms22147664/s1, Figure S1: The phylogenetic tree based on data by Springer et al. (Springer, M.S., Stanhope, M.J., Madsen, O., de Jong, W.W. Molecules consolidate the placental mammal tree. *Trends Ecol Evol*. **2004**, 19, 430–438.) and Brace et al. (Brace, S., Thomas, J.A., Dalén, L., Burger, J., MacPhee, R.D., Barnes, I., Turvey, S.T. Evolutionary History of the Nesophontidae, the Last Unplaced Recent Mammal Family. *Mol Biol Evol*. **2016**, 33, 3095–3103).
