# Supplementary material for: Effects of Brain Size on Adult Neurogenesis in Shrews
Source: Int J Mol Sci. 2021 Jul 17;22(14):7664. doi: 10.3390/ijms22147664 (PMC8303847; doi:10.3390/ijms22147664)
Supplement: Supplementary file 1 [file ijms-22-07664-s001.zip › ijms-1266692-supplementary.pdf]

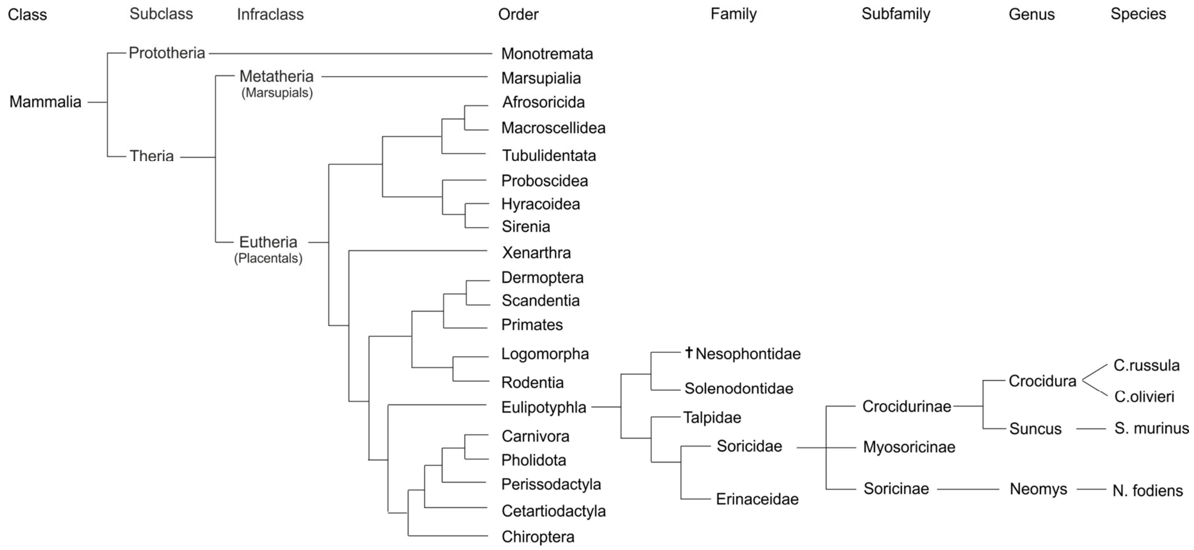

Figure S1. The phylogenetic tree based on data by Springer et al. (Springer, M.S., Stanhope, M.J., Madsen, O., de Jong, W.W. Molecules consolidate the placental mammal tree. Trends Ecol Evol. 2004, 19, 430-438.) and Brace et al. (Brace, S., Thomas, J.A., Dalén, L., Burger, J., MacPhee, R.D., Barnes, I., Turvey, S.T. Evolutionary History of the Nesophontidae, the Last Unplaced Recent Mammal Family. Mol Biol Evol. 2016, 33, 3095-3103).
